# Sensory impairment and cognitive-state transitions in middle-aged and older adults: a multistate Markov analysis of the health and retirement study

**DOI:** 10.3389/fnagi.2026.1913857

**Published:** 2026-09-07

**Authors:** Zhongxing Liu, Lichen Wang, Peng Guo

**Affiliations:** 1 Department of Traditional Chinese Medicine, Chengdu Integrated TCM and Western Medicine Hospital, Chengdu, China; 2 School of Clinical Medicine, Chengdu University of Traditional Chinese Medicine, Chengdu, China; 3 Department of Traditional Chinese Medicine, Chengdu Second People’s Hospital, Chengdu, China

**Keywords:** cognitive ageing, cognitive impairment no dementia, health and retirement study, multistate Markov model, sensory impairment

## Abstract

**Background:**

Sensory impairment has been associated with cognitive decline and dementia. However, endpoint-based longitudinal analyses often cannot determine whether sensory impairment is related to early cognitive deterioration, reduced cognitive recovery, dementia progression, or mortality-related study exit.

**Methods:**

We analyzed 2004–2022 Health and Retirement Study data using a continuous-time multistate Markov model. Four states were defined: normal cognition, cognitive impairment no dementia (CIND), dementia, and death. The analytic cohort included 18,292 participants, and 16,083 were included in the primary complete-case adjusted model. Baseline self-reported sensory status was classified as no impairment, visual impairment only, hearing impairment only, or dual sensory impairment. Primary models adjusted for age, sex, education, wealth, depressive symptoms, and chronic disease count; sensitivity analyses included time-varying and lagged exposures, inverse-probability weighting, and expanded confounding adjustment.

**Results:**

Compared with no sensory impairment, visual impairment only, hearing impairment only, and dual sensory impairment were associated with faster transitions from normal cognition to CIND. The corresponding HRs were 1.18 (95% CI, 1.10–1.27), 1.16 (95% CI, 1.07–1.25), and 1.37 (95% CI, 1.26–1.50). Visual impairment only and dual sensory impairment were also associated with slower transitions from CIND to normal cognition, with HRs of 0.79 (95% CI, 0.72–0.86) and 0.84 (95% CI, 0.75–0.94), respectively. Associations with CIND-to-dementia progression and mortality-related transitions were less consistent. Time-varying, lagged, weighted, and expanded-confounding analyses supported the same early-transition pattern.

**Conclusion:**

Sensory impairment, particularly dual sensory impairment, was most consistently associated with early cognitive deterioration and reduced cognitive recovery. It was not consistently associated with higher dementia progression or mortality transition rates. Death-inclusive multistate modeling may help identify where potentially modifiable sensory impairment is related to cognitive ageing trajectories.

## Introduction

Dementia and sensory impairment are increasingly common as populations age, and both impose substantial public health burdens. The World Health Organization estimated that approximately 57 million people were living with dementia in 2021, with nearly 10 million new cases each year; GBD projections further suggest that the global number of people living with dementia will rise substantially by 2050 (GBD 2019 Dementia Forecasting Collaborators, 2022; World Health Organization, 2025). Sensory impairment is similarly widespread. By 2050, nearly 2.5 billion people are projected to have some degree of hearing loss. At least 2.2 billion people globally have near or distance vision impairment, including at least 1 billion cases that are preventable or remain unaddressed (World Health Organization, 2019, 2026; GBD 2019 Blindness and Vision Impairment Collaborators and Vision Loss Expert Group of the Global Burden of Disease Study, 2021; GBD 2019 Hearing Loss Collaborators, 2021). Sensory impairment is therefore not only a major source of late-life functional limitation, but also a plausible intervention point in cognitive ageing and dementia prevention.

Accumulating evidence links hearing loss to cognitive decline, cognitive impairment, and dementia. Vision impairment has also been associated with adverse cognitive outcomes in older adults (Lin et al., 2011; Loughrey et al., 2018; Cao et al., 2022; Livingston et al., 2024). Hearing and vision problems frequently co-occur, and dual sensory impairment may confer particularly high dementia risk (Kuo et al., 2021; Hwang et al., 2022). Evidence from United States ageing cohorts, including the Health and Retirement Study (HRS) and HRS-ADAMS, further suggests that self-reported or objectively measured visual, hearing, and dual sensory impairment are associated with cognitive decline, incident cognitive impairment no dementia (CIND), dementia, or Alzheimer’s disease (Maharani et al., 2019; Ge et al., 2020; Li et al., 2024).

However, most studies of sensory impairment and cognition have treated cognitive decline, incident cognitive impairment, or dementia as single endpoints. Such designs can show whether sensory impairment predicts a later cognitive outcome, but they cannot determine which part of the cognitive ageing process is most affected. Older adults may move dynamically between normal cognition, CIND, dementia, and death. Some recover from cognitive impairment, whereas others progress to dementia or die before dementia is observed. Death is especially important in older cohorts because it is a competing absorbing state, not merely ordinary loss to follow-up. Multistate models can estimate transition-specific associations across cognitive states while incorporating death into the same disease-process framework (Putter et al., 2006; Jackson, 2011).

Previous multistate studies have examined education, multimorbidity patterns, and sensory domains such as olfaction in relation to transitions across cognitive states and death (Reuser et al., 2011; Robitaille et al., 2018; Knight et al., 2023; Valletta et al., 2023). For example, a population-based SNAC-K study modelled transitions among normal cognition, CIND, dementia, and death. It found that a multimorbidity pattern including sensory impairment and cancer was associated with lower recovery from CIND to normal cognition (Valletta et al., 2023). These studies provide important methodological precedent. Yet they have not estimated transition-specific associations of visual impairment, hearing impairment, and dual sensory impairment as distinct exposures in HRS.

Stage-specific associations are plausible within cognitive reserve and sensory-deprivation frameworks. Cognitive reserve describes the capacity to maintain function despite age-related brain change or pathology (Stern, 2012). Intact vision and hearing may support environmental stimulation, communication, reading, navigation, and compensatory processing, whereas sensory loss may increase cognitive load and reduce opportunities to maintain reserve (Lindenberger and Baltes, 1994; Wayne and Johnsrude, 2015; Whitson et al., 2018). These pathways may be most detectable before advanced neurodegenerative pathology dominates clinical progression, providing a rationale for examining whether sensory impairment is more strongly related to early cognitive deterioration and recovery than to later dementia transitions.

We therefore used HRS data from 2004 to 2022 to examine how baseline visual, hearing, and dual sensory impairment were associated with transitions among normal cognition, CIND, dementia, and death. We hypothesized that sensory impairment, particularly dual sensory impairment, would be associated with higher rates of cognitive deterioration, lower rates of recovery to a less impaired state, and higher transition rates to death, while allowing the magnitude of these associations to differ across transitions. Normal cognition to CIND and CIND to normal cognition were prespecified as the primary interpretive transitions; CIND to dementia progression, reverse classification transitions, and mortality-related transitions were assessed as secondary or exploratory outcomes.

## Methods

### Study design and data source

We conducted a longitudinal cohort analysis using data from the Health and Retirement Study (HRS), an ongoing nationally representative study of United States adults aged 50 years or older and their spouses (Sonnega et al., 2014; Health and Retirement Study, 2025). We used HRS waves 7–16, corresponding to biennial assessments from 2004 to 2022. Demographic, health, economic, and interview-status variables were obtained from the RAND HRS Longitudinal File and linked to HRS sensory measures and Langa-Weir cognitive classification files (Crimmins et al., 2011; Langa et al., 2025; RAND Center for the Study of Aging, 2025).

Participants were eligible if they were alive at wave 7, aged 50 years or older, had a baseline cognitive state classified as normal cognition, cognitive impairment no dementia (CIND), or dementia, had non-missing baseline sensory status, sex, and education, and had longitudinal state histories available for multistate model construction. The analytic cohort included 18,292 participants. The primary adjusted model was restricted to participants with complete data for all model covariates and included 16,083 participants. We summarized covariate missingness and compared baseline characteristics between participants included in and excluded from the complete-case adjusted model to assess potential selection differences.

### Sensory impairment

Baseline sensory impairment was defined using self-rated vision and hearing at HRS wave 7. The 2004 HRS questions asked participants to rate eyesight while using glasses or corrective lenses as usual and hearing while using a hearing aid as usual. The exposure therefore reflects perceived functional sensory status under usual correction or assistance, rather than uncorrected acuity or audiometric thresholds. Responses indicating fair or poor vision or hearing, blindness, or deafness were classified as impairment. Responses of excellent, very good, or good were classified as no impairment. Participants were categorized into four mutually exclusive exposure groups: no sensory impairment, visual impairment only, hearing impairment only, and dual sensory impairment.

In sensitivity analyses, we repeated the analyses using time-varying sensory status, lagged sensory status, and a stricter impairment definition based on poor vision or hearing, blindness, or deafness. Time-varying sensory status was updated at each available HRS wave using wave-specific self-rated vision and hearing, and a lagged exposure model used sensory status from the previous wave. These measures capture changes in perceived function under usual correction or assistance but cannot distinguish natural progression from treatment, device uptake, or measurement variation.

### Cognitive and mortality states

Cognitive status was defined using the Langa-Weir classification, which categorizes HRS participants as having normal cognition, CIND, or dementia using survey-based cognitive measures and proxy information when applicable (Crimmins et al., 2011; Langa et al., 2025). The first death record was retained, and records after death were excluded. The four states in the primary multistate model were normal cognition, CIND, dementia, and death.

### Covariates

Primary models adjusted for baseline age, sex, education, wealth tertile, depressive symptoms, and chronic disease count. Education was modelled using the five RAND HRS education categories. Depressive symptoms were measured using the HRS CES-D score. Chronic disease count was calculated as the sum of self-reported hypertension, diabetes, cancer, lung disease, heart disease, stroke, psychiatric illness, and arthritis. An expanded-confounding sensitivity model additionally adjusted for baseline current smoking, any alcohol use, weekly moderate or vigorous physical activity, and regular religious participation (at least two or three times per month) as an available but partial proxy for social engagement. We refitted the core model in the same complete-case sample used for the expanded model to separate covariate-adjustment effects from sample restriction. Baseline differences in age and education across sensory groups were assessed using one-way analysis of variance and Pearson’s chi-square test, respectively.

### Multistate model

We fitted continuous-time multistate Markov models for panel-observed state histories using the R package msm (Jackson, 2011). The primary transition structure allowed bidirectional transitions between normal cognition and CIND, transitions between CIND and dementia, and transitions from each living cognitive state to death. Death was treated as an absorbing state.

Direct transitions from normal cognition to dementia were not parameterized in the primary model. This structure reflects the conceptual role of CIND as an intermediate cognitive state and recognizes that apparent normal-to-dementia changes across two-year survey intervals may include unobserved intermediate impairment. Observed normal-to-dementia intervals were therefore handled within the interval-censored continuous-time process rather than interpreted as instantaneous direct clinical transitions. Observed transition counts underlying the panel-state histories are provided in Supplementary Table S1.

The dementia-to-CIND transition was allowed in the primary model to accommodate observed fluctuation in survey-based cognitive classification. This transition was interpreted cautiously and was not considered evidence of clinical dementia reversal. Hazard ratios were estimated for each sensory impairment group relative to the no-impairment group within each allowed transition. Model-based state occupation probabilities were estimated for participants starting from normal cognition at representative covariate values and summarized at 5, 10, and 15 years after baseline. To improve clinical interpretability, we additionally estimated restricted expected time in normal cognition, CIND, dementia, and death by integrating model-based state occupation probabilities over 5-, 10-, and 15-year horizons.

### Sensitivity and supplementary analyses

We conducted multiple sensitivity analyses to assess the robustness of the main transition-specific findings. These included a time-varying sensory exposure model, a lagged sensory exposure model, restriction to participants with normal cognition at baseline, exclusion of participants with dementia at baseline, stricter sensory impairment definitions, an absorbing dementia assumption, refined death timing using available death-year information, and an expanded transition-structure check allowing normal cognition-to-dementia intervals. We also fitted a complete-case inverse-probability weighted model to assess potential selection into the complete-case adjusted sample, an observed spell-duration adjusted model, and the expanded-confounding model described above. The primary time-homogeneous model was compared with a piecewise model to assess whether transition intensities varied over follow-up.

The two prespecified primary interpretive transitions were normal cognition to CIND and CIND to normal cognition. Dementia progression, mortality transitions, model-structure checks, and subgroup analyses were treated as secondary or exploratory.

This was an observational, model-based analysis. Hazard ratios should therefore be interpreted as transition-specific associations rather than causal effects. Primary models were not survey-weighted because the continuous-time multistate likelihood was used to estimate model-based transition intensities. Population generalizability was addressed through the HRS sampling frame, covariate adjustment, and explicit discussion of this limitation.

## Results

### Study population

**Figure 1:**
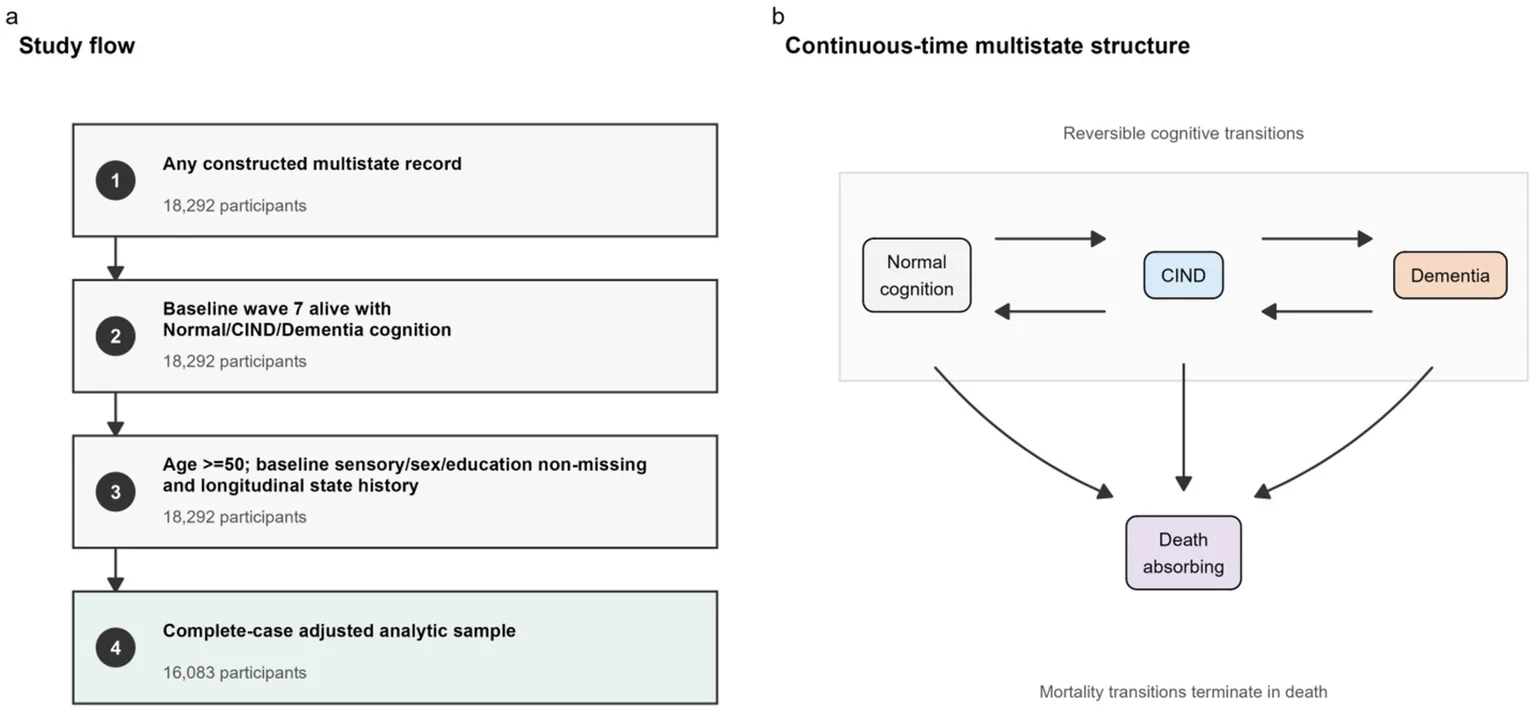
Study flow and multistate model structure. **(a)** Flow of HRS participants included in the analytic sample. The complete-case adjusted HRS sample included 16,083 participants. **(b)** Continuous-time multistate Markov model structure. The model allowed transitions between normal cognition and CIND, between CIND and dementia, and from each living cognitive state to death. Death was treated as an absorbing state.

The analytic cohort included 18,292 HRS participants with at least one observed state record contributing to the multistate analysis. After restriction to the complete-case adjusted sample, 16,083 participants were included in the primary multistate Markov model (Figure 1). The main sources of covariate missingness were wealth tertile and depressive symptoms. Baseline characteristics of participants included in and excluded from the complete-case adjusted model are shown in Supplementary Tables S2, S3.

In the complete-case adjusted sample, 10,628 participants had no sensory impairment, 2,104 had visual impairment only, 2,035 had hearing impairment only, and 1,316 had dual sensory impairment. Age differed across sensory groups (omnibus *p* < 0.001), as did the distribution of education (omnibus *p* < 0.001); both variables were included in every adjusted multistate model. Compared with participants without sensory impairment, those with sensory impairment were older and had a higher burden of depressive symptoms and chronic conditions (Table 1). The dual sensory impairment group had the oldest mean age and the highest prevalence of baseline cognitive impairment. Normal cognition at baseline was present in 85.5% of participants without sensory impairment, compared with 68.3, 75.3, and 59.9% of those with visual, hearing, and dual impairment, respectively.

**Table 1:** Baseline characteristics by sensory impairment group in the complete-case adjusted sample.

Baseline sensory group	*N*	Age, mean (SD)	Female, *n* (%)	Lower education, *n* (%)	CESD, mean (SD)	Chronic conditions, mean (SD)	Normal cognition, *n* (%)	CIND, *n* (%)	Dementia, *n* (%)
No impairment	10,628	66.0 (9.8)	6,585 (62.0)	1,673 (15.7)	1.1 (1.7)	1.6 (1.3)	9,084 (85.5)	1,273 (12.0)	271 (2.5)
Visual only	2,104	68.4 (11.1)	1,414 (67.2)	755 (35.9)	2.3 (2.3)	2.3 (1.5)	1,436 (68.3)	511 (24.3)	157 (7.5)
Hearing only	2035	70.9 (10.5)	841 (41.3)	470 (23.1)	1.5 (1.9)	2.1 (1.4)	1,532 (75.3)	414 (20.3)	89 (4.4)
Dual impairment	1,316	72.1 (11.5)	684 (52.0)	527 (40.0)	2.6 (2.3)	2.6 (1.6)	788 (59.9)	405 (30.8)	123 (9.3)

### Primary transition-specific associations

**Figure 2:**
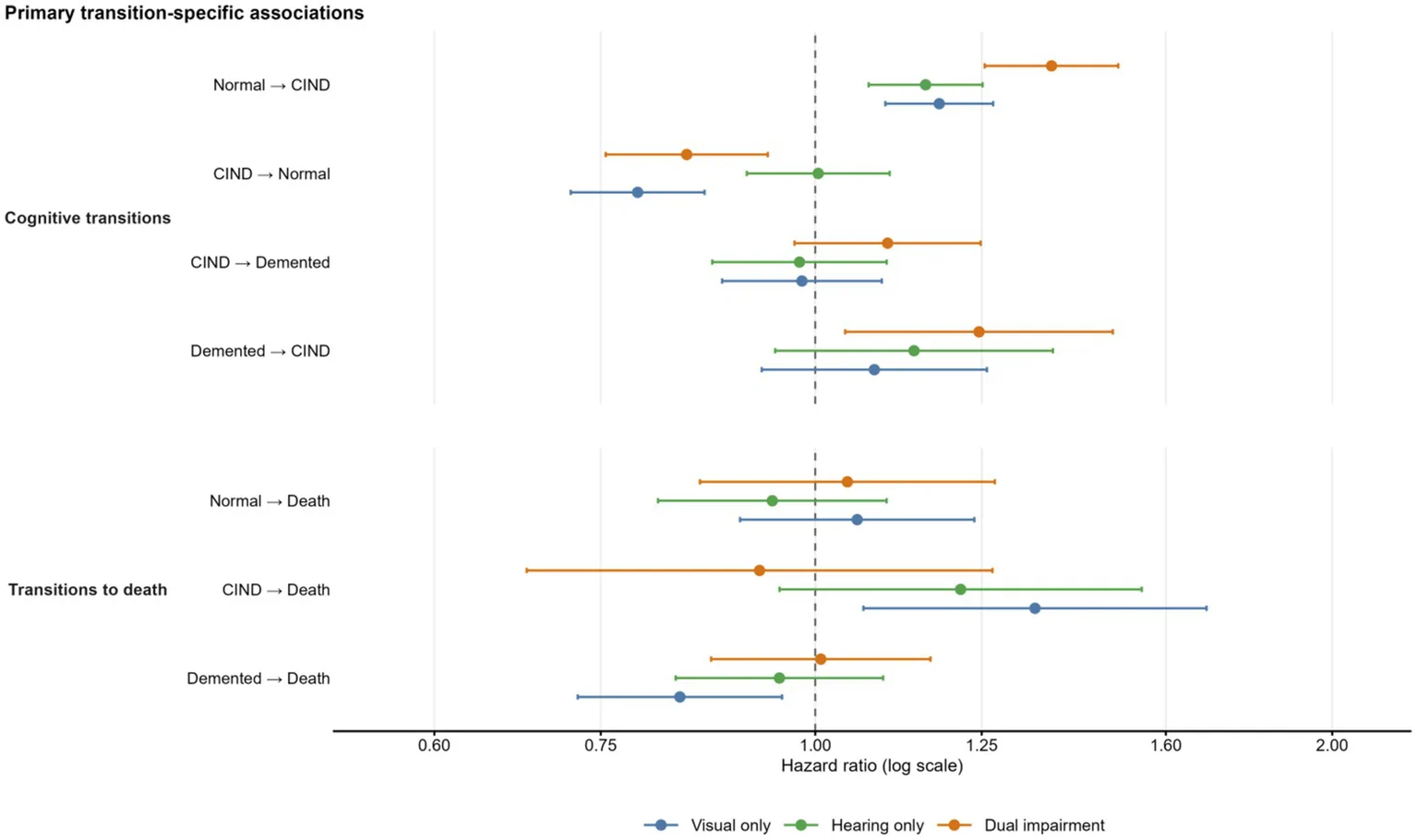
Primary HRS transition-specific hazard ratios. Forest plots show transition-specific hazard ratios and 95% confidence intervals for visual impairment only, hearing impairment only, and dual sensory impairment compared with no sensory impairment. Estimates were obtained from the primary adjusted continuous-time multistate Markov model. The model adjusted for age, sex, education, wealth tertile, depressive symptoms, and chronic disease count. The vertical dashed line indicates *HR* = 1.

In the primary adjusted model, all three sensory impairment groups had higher transition rates from normal cognition to CIND than the no-impairment group (Figure 2 and Table 2). The HRs were 1.18 (95% CI, 1.10–1.27) for visual impairment only, 1.16 (95% CI, 1.07–1.25) for hearing impairment only, and 1.37 (95% CI, 1.26–1.50) for dual sensory impairment.

**Table 2:** Primary transition-specific hazard ratios by baseline sensory impairment group.

Transition	Visual only	Hearing only	Dual impairment
Normal → CIND	1.18 (1.10–1.27)	1.16 (1.07–1.25)	1.37 (1.26–1.50)
Normal → Death	1.06 (0.90–1.24)	0.94 (0.81–1.10)	1.04 (0.86–1.27)
CIND → Normal	0.79 (0.72–0.86)	1.00 (0.91–1.10)	0.84 (0.75–0.94)
CIND → Dementia	0.98 (0.88–1.09)	0.98 (0.87–1.10)	1.10 (0.97–1.25)
CIND → Death	1.34 (1.07–1.69)	1.22 (0.95–1.55)	0.93 (0.68–1.27)
Dementia → CIND	1.08 (0.93–1.26)	1.14 (0.95–1.38)	1.25 (1.04–1.49)
Dementia → Death	0.83 (0.73–0.96)	0.95 (0.83–1.10)	1.01 (0.87–1.17)

Hazard ratios compare each sensory impairment group with the no sensory impairment group. The primary model adjusted for age, sex, education, wealth tertile, depressive symptoms, and chronic disease count.

Visual impairment only and dual sensory impairment were also associated with lower transition rates from CIND back to normal cognition. The HRs were 0.79 (95% CI, 0.72–0.86) for visual impairment only and 0.84 (95% CI, 0.75–0.94) for dual sensory impairment. Hearing impairment only was not associated with this recovery transition (HR, 1.00; 95% CI, 0.91–1.10).

Associations with progression from CIND to dementia were less consistent. The HRs were 0.98 (95% CI, 0.88–1.09) for visual impairment only, 0.98 (95% CI, 0.87–1.10) for hearing impairment only, and 1.10 (95% CI, 0.97–1.25) for dual sensory impairment. In the full transition-specific output, dual sensory impairment was associated with a higher transition rate from dementia to CIND (HR, 1.25; 95% CI, 1.04–1.49). This reverse transition was included to accommodate fluctuation in survey-based cognitive classification and was not interpreted as clinical reversal of dementia.

Mortality-related transitions showed weaker and less uniform associations. Visual impairment only was associated with a higher CIND-to-death transition rate (HR, 1.34; 95% CI, 1.07–1.69) but with a lower dementia-to-death transition rate (HR, 0.83; 95% CI, 0.73–0.96). The latter direction was counterintuitive. No other sensory group showed a statistically clear association with mortality-related transitions, and mortality findings were treated as secondary and interpreted cautiously.

### Predicted state occupation probabilities

**Figure 3:**
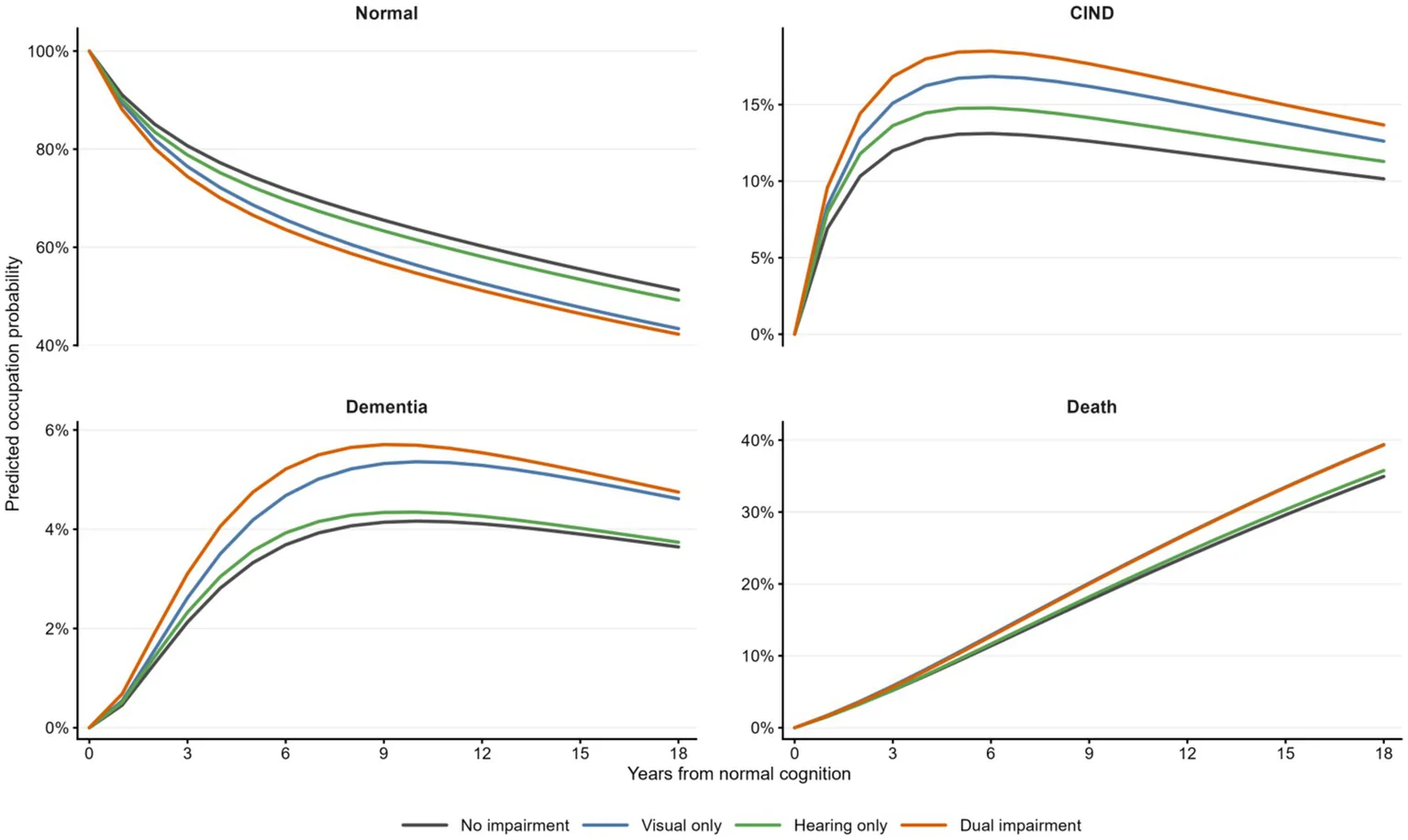
Predicted cognitive and mortality state occupation from normal cognition. Model-based predicted state occupation probabilities are shown for participants starting from normal cognition, stratified by baseline sensory impairment group. Probabilities were estimated from the primary adjusted HRS multistate model at representative covariate values. Curves show the predicted probability of occupying normal cognition, CIND, dementia, or death over follow-up.

Model-based state occupation probabilities for participants starting from normal cognition showed graded differences across sensory impairment groups (Figure 3 and Table 3). At 10 years, the predicted probability of remaining in normal cognition was 63.7% for participants without sensory impairment, 56.3% for visual impairment only, 61.5% for hearing impairment only, and 54.7% for dual sensory impairment. The corresponding predicted probabilities of CIND were 12.4, 15.8, 13.8, and 17.2%. Dual sensory impairment also had the highest predicted dementia probability at 10 years (5.7%).

**Table 3:** Predicted state occupation probabilities from normal cognition.

Baseline sensory group	Years	Normal	CIND	Dementia	Death
No impairment	5	74.3%	13.1%	3.3%	9.3%
No impairment	10	63.7%	12.4%	4.2%	19.8%
No impairment	15	55.5%	11.0%	3.9%	29.6%
Visual only	5	68.6%	16.7%	4.2%	10.5%
Visual only	10	56.3%	15.8%	5.4%	22.5%
Visual only	15	47.7%	13.8%	5.0%	33.5%
Hearing only	5	72.2%	14.8%	3.6%	9.5%
Hearing only	10	61.5%	13.8%	4.3%	20.3%
Hearing only	15	53.4%	12.2%	4.0%	30.3%
Dual impairment	5	66.5%	18.4%	4.7%	10.3%
Dual impairment	10	54.7%	17.2%	5.7%	22.4%
Dual impairment	15	46.5%	15.0%	5.2%	33.4%

At 15 years, the predicted probability of remaining in normal cognition was 55.5% in the no-impairment group and 46.5% in the dual sensory impairment group. The predicted probability of death at 15 years was 29.6% for no sensory impairment, 33.5% for visual impairment only, 30.3% for hearing impairment only, and 33.4% for dual sensory impairment. Descriptive observed state occupation over survey years is shown in Supplementary Figure S1.

Restricted expected-time estimates showed the same pattern in years rather than probabilities (Supplementary Figure S2 and Supplementary Table S4). Over 10 years, participants without sensory impairment were expected to spend 7.64 years in normal cognition and 1.14 years with CIND. Compared with no sensory impairment, visual impairment only, hearing impairment only, and dual sensory impairment were associated with 0.51, 0.19, and 0.68 fewer expected years in normal cognition, respectively, and 0.31, 0.15, and 0.46 additional expected years with CIND. Over 15 years, dual sensory impairment was associated with 1.14 fewer expected years in normal cognition and 0.68 additional expected years with CIND.

### Sensitivity analyses

**Figure 4:**
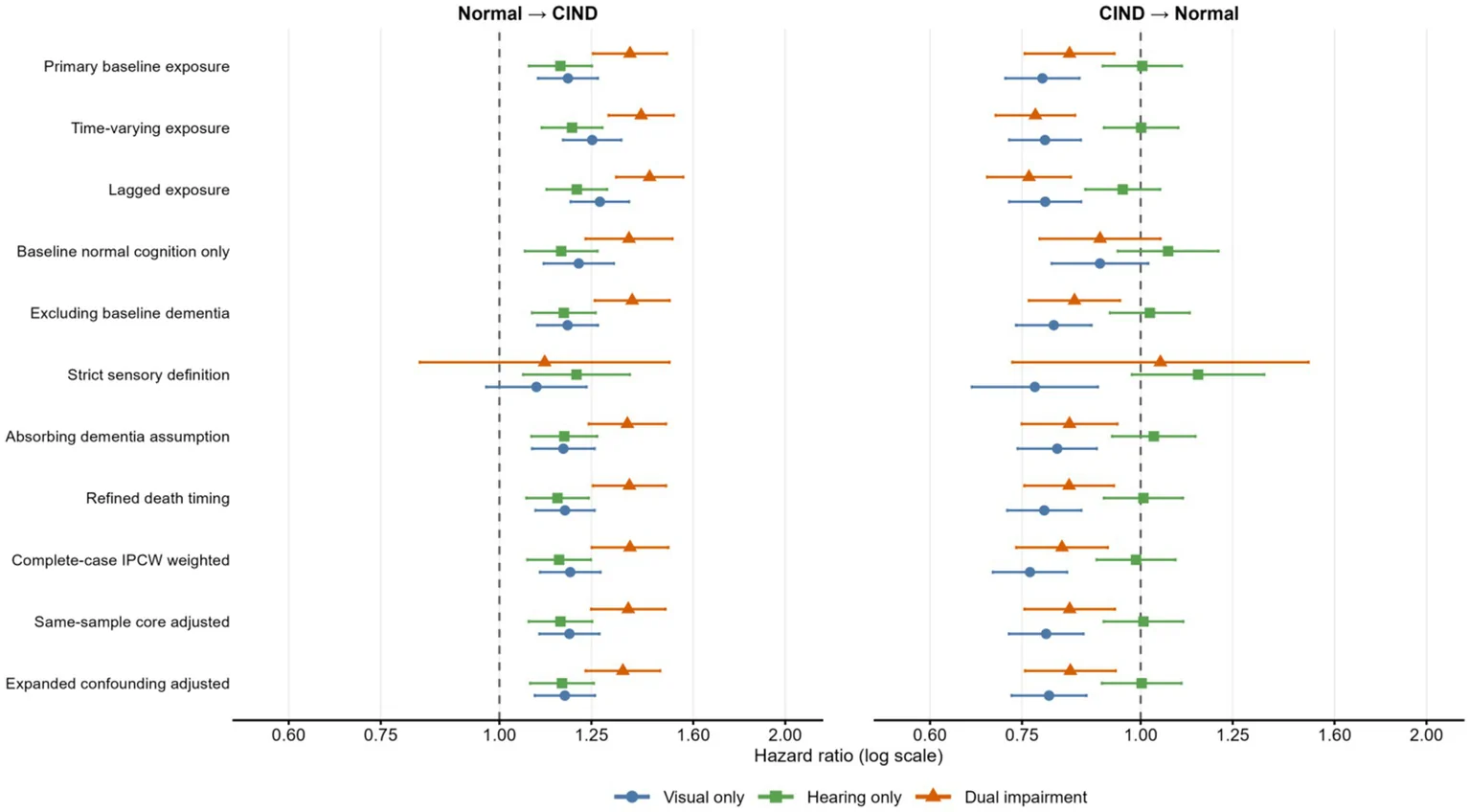
Robustness of early cognitive transition findings. Forest plots summarize hazard ratios and 95% confidence intervals for normal cognition to CIND and CIND to normal cognition across the primary model and selected sensitivity analyses. Models included the primary baseline exposure model, time-varying exposure model, lagged exposure model, baseline-normal restriction, exclusion of baseline dementia, strict sensory impairment definition, absorbing dementia assumption, refined death timing, complete-case inverse-probability weighting, a core model refitted in the expanded-model sample, and expanded adjustment for smoking, alcohol use, physical activity, and regular religious participation. Blue circles, green squares, and orange triangles denote visual impairment only, hearing impairment only, and dual sensory impairment, respectively. The vertical dashed line indicates *HR* = 1.

Sensitivity analyses supported the stability of the early-transition findings (Figure 4 and Supplementary Table S5). In models using time-varying sensory status, visual impairment only, hearing impairment only, and dual sensory impairment were each associated with higher transition rates from normal cognition to CIND, with HRs of 1.25 (95% CI, 1.17–1.34), 1.19 (95% CI, 1.11–1.28), and 1.41 (95% CI, 1.30–1.53), respectively. Visual impairment only and dual sensory impairment were also associated with lower transition rates from CIND to normal cognition (HR, 0.79; 95% CI, 0.73–0.86; and HR, 0.77; 95% CI, 0.70–0.85, respectively). Lagged exposure models yielded similar estimates; additional details on these models and the time-varying sensory distribution are provided in Supplementary Figure S3 and Supplementary Tables S6–S8.

Findings for CIND-to-dementia progression were less consistent across model specifications, although dual sensory impairment was associated with higher CIND-to-dementia transition rates in the time-varying exposure model (HR, 1.23; 95% CI, 1.10–1.37) and lagged exposure model (HR, 1.27; 95% CI, 1.13–1.42) (Supplementary Figure S4). In the inverse-probability weighted analysis, the HRs for normal cognition to CIND were 1.19, 1.16, and 1.37 for visual, hearing, and dual impairment, respectively; the corresponding HRs for CIND to normal cognition were 0.76, 0.99, and 0.83 (Supplementary Table S6). In the expanded transition-structure check that allowed normal cognition-to-dementia intervals, estimates for the two primary early transitions were materially unchanged, although the model produced a non-positive Hessian and was interpreted only as a structural check (Supplementary Table S9). The piecewise time-homogeneity assessment suggested variation in transition intensities over follow-up (Supplementary Table S10).

Exploratory age-stratified transition-specific logistic analyses suggested stronger associations before age 75. Among participants aged younger than 75 years, visual, hearing, and dual impairment were associated with normal cognition-to-CIND odds ratios of 1.28, 1.17, and 1.50, respectively, whereas corresponding estimates among those aged 75 years or older were 1.08, 1.05, and 1.14 and their confidence intervals included unity. Visual and dual impairment were also associated with lower CIND-to-normal odds among those younger than 75 years, but not among those aged 75 years or older (Supplementary Table S11). Across education strata, dual impairment remained associated with higher normal cognition-to-CIND and lower CIND-to-normal odds. These subgroup models were exploratory, and differences in stratum-specific statistical significance do not by themselves establish interaction.

An expanded-confounding sensitivity analysis included 15,936 participants. After additional adjustment for current smoking, alcohol use, physical activity, and regular religious participation, the HRs (95% CIs) for normal cognition to CIND were 1.17 (1.09–1.26), 1.16 (1.08–1.26), and 1.35 (1.23–1.48); the corresponding HRs (95% CIs) for CIND to normal cognition were 0.80 (0.73–0.88), 1.00 (0.91–1.10), and 0.84 (0.76–0.94) for visual, hearing, and dual impairment, respectively. Estimates were similar to those from the core-adjusted model restricted to the same sample (Supplementary Table S12), indicating that these additional measured covariates did not materially change the early-transition pattern. Religious participation was only a partial proxy for social engagement.

## Discussion

In this death-inclusive multistate analysis of HRS participants, the prespecified hypotheses were partially supported. As hypothesized, visual, hearing, and dual sensory impairment were each associated with faster transition from normal cognition to CIND, and visual and dual impairment were associated with slower transition from CIND back to normal cognition. Dual impairment generally showed the largest early-transition association. However, the hypotheses of uniformly higher later dementia progression and mortality transition rates were not supported; these associations were weaker, heterogeneous, or counterintuitive. The results therefore localize the most consistent associations to early cognitive deterioration and recovery rather than to uniform acceleration across all later transitions.

Predicted state occupation probabilities translated these transition-specific associations into absolute differences. Among participants starting from normal cognition, dual sensory impairment was associated with a 9.0 percentage-point lower predicted probability of remaining cognitively normal at 10 years. It was also associated with a 4.8 percentage-point higher predicted probability of CIND compared with no sensory impairment. These differences are modest at the individual level, but they may be clinically and public-health relevant in older populations because CIND is common, dynamic, and potentially more amenable to prevention than established dementia.

These findings are consistent with prior evidence linking sensory impairment to adverse cognitive outcomes. Longitudinal studies and meta-analyses have associated hearing loss with cognitive decline, cognitive impairment, and dementia (Lin et al., 2011; Loughrey et al., 2018; Livingston et al., 2024). Vision impairment has also been linked to poorer cognitive outcomes in older adults (Rogers and Langa, 2010; Cao et al., 2022). In HRS, Maharani et al. (2019) reported that self-reported single or dual sensory impairment was associated with cognitive decline, incident possible CIND, and probable dementia. Li et al. (2024) further found associations of visual, hearing, and dual sensory impairment with 10-year dementia and Alzheimer’s disease risk. HRS-ADAMS analyses using objective hearing and vision measures also support an association between sensory loss and cognitive decline (Ge et al., 2020). These studies established that sensory impairment predicts cognitive endpoints. The present study extends that evidence by showing that the most consistent associations were concentrated in normal-to-CIND deterioration and CIND-to-normal recovery.

The findings also build on prior cognitive multistate studies. Reuser et al. (2011) used HRS multistate life-table methods to examine cognitive impairment, recovery, and death, although their main exposures were education, body mass index, and smoking. Robitaille et al. (2018) examined education in relation to cognitive-state and death transitions across multiple longitudinal studies. Knight et al. (2023) incorporated olfaction into a multistate framework for mild cognitive impairment, dementia, and mortality. The closest methodological precedent is the SNAC-K multimorbidity-pattern study by Valletta et al. (2023), which modelled normal cognition, CIND, dementia, and death. That study found that a multimorbidity pattern including sensory impairment and cancer was associated with lower recovery from CIND to normal cognition. Our study complements this work by estimating transition-specific associations for visual impairment, hearing impairment, and dual sensory impairment as distinct exposures in HRS.

The concentration of associations in early transitions is compatible with cognitive reserve and sensory-deprivation frameworks. Before established dementia, intact vision and hearing may help sustain reserve through communication, reading, mobility, navigation, and environmental stimulation, whereas sensory loss may increase cognitive load and reduce stimulation (Crimmins et al., 2011; Langa et al., 2025; RAND Center for the Study of Aging, 2025); cognitive reserve theory provides a complementary explanation (Stern, 2012). Shared vascular, neurodegenerative, and frailty-related causes may also contribute, and hearing loss has been related to neural and brain-volume changes (Lin et al., 2014; Wayne and Johnsrude, 2015; Griffiths et al., 2020). Social isolation and depressive symptoms are additional plausible pathways (Mick et al., 2014; Dawes et al., 2015; Kuiper et al., 2015; Yorgason et al., 2022). Once dementia is established, underlying neuropathology and broader health burden may dominate subsequent progression, potentially weakening the relative contribution of sensory status. These mechanisms fit the observed pattern but were not directly tested and should not be interpreted causally.

The absence of an association between hearing impairment only and CIND-to-normal recovery, in contrast to visual and dual impairment, may reflect modality-specific pathways, measurement error, or chance. Vision supports reading, visuospatial navigation, mobility, and other daily activities that provide cognitive stimulation, and objective visual measures have been associated with decline across several cognitive domains (Varadaraj et al., 2021). By contrast, self-rated hearing has limited sensitivity to audiometric loss, particularly among adults with cognitive impairment (Kim et al., 2021), which could attenuate hearing-specific associations. Nevertheless, this observational analysis cannot establish that visual input is intrinsically more important for cognitive recovery than hearing, and the heterogeneity requires replication.

Dual sensory impairment may be especially important. When both visual and hearing channels are impaired, compensation through the unaffected sensory channel is limited. Restrictions on communication, mobility, and environmental engagement may therefore be greater than with either impairment alone. Prior studies have associated dual sensory impairment with dementia, serious cognitive impairment, and faster cognitive decline (Maharani et al., 2018, 2019; Ge et al., 2020; Kuo et al., 2021; Fuller-Thomson et al., 2022; Hwang et al., 2022; Li et al., 2024). In the present study, dual sensory impairment showed the largest and most consistent association with transition from normal cognition to CIND. This pattern remained directionally stable across multiple sensitivity analyses. These findings support considering dual sensory impairment as a combined exposure in cognitive-risk assessment, rather than treating visual and hearing impairment only as isolated deficits.

A key methodological strength of this study is the inclusion of death as an absorbing state. In older cohorts, death is not ordinary attrition because it terminates the possibility of subsequent cognitive transitions. Ignoring death may distort interpretation of dementia-related pathways, particularly when exposures are also associated with survival. Multistate models can estimate transitions between cognitive states and transitions from living cognitive states to death within the same framework (Putter et al., 2006; Jackson, 2011; Reuser et al., 2011; Robitaille et al., 2018; Knight et al., 2023; Valletta et al., 2023). In this analysis, mortality transitions were not the most stable substantive findings. Their main value was to reduce the risk of treating mortality-related exit as non-informative loss to follow-up.

The exploratory age-stratified results also require cautious interpretation. Stronger associations before age 75 could reflect a stage at which sensory input and compensatory resources remain more influential, whereas at older ages greater neurodegenerative and multimorbidity burdens may dominate cognitive transitions. Selective survival, exposure misclassification, and lower precision in the older stratum are additional explanations. Because the subgroup analyses were not formal interaction tests, the apparent attenuation at age 75 years or older should be viewed as hypothesis-generating rather than evidence of a definitive age threshold.

The mortality findings should not be interpreted as protective or causal. Transition-specific hazard ratios are conditional on occupying CIND or dementia, and the sensory groups that reach those states may differ because of earlier transitions, survival, and care. Smaller numbers in the dual-impairment and dementia strata, selective survival, exposure or state misclassification, residual confounding, and multiple secondary comparisons may therefore produce unstable or paradoxical estimates. The inverse-probability weighted analysis addressed observed selection into the complete-case sample but not state-dependent selection or unmeasured confounding. We consequently regard the elevated visual-only CIND-to-death estimate and the lower visual-only dementia-to-death estimate as exploratory.

This study also has limitations. First, vision and hearing were self-rated under usual correction or assistance rather than assessed using acuity or audiometry. Self-report can disagree with objective impairment, and unrecognized hearing loss may be especially common among people with cognitive impairment (Kim et al., 2021). Time-varying and lagged analyses captured reported change but could not distinguish sensory progression from treatment, device uptake, or measurement variation. Second, the Langa-Weir classification is survey based rather than a clinical diagnosis (Crimmins et al., 2011); adjacent-state fluctuation is possible, and dementia-to-CIND transitions should not be interpreted as clinical reversal. Third, the primary structure did not estimate a separate direct normal cognition-to-dementia transition; the expanded direct-transition model was not stable enough for formal inference. Fourth, excluded participants were older and had greater cognitive and sensory burden. Inverse-probability weighted estimates were similar to the primary results, but selection bias cannot be excluded and its direction cannot be established from these data. Fifth, models were not survey weighted and should be interpreted as model-based associations in HRS rather than exact population estimates. Finally, the expanded model adjusted for smoking, alcohol use, physical activity, and regular religious participation, but the latter is an incomplete proxy for social engagement and residual confounding remains possible. The time-homogeneity assessment also suggested that transition intensities may vary over follow-up, so primary estimates represent average transition-specific associations over the study period.

Overall, this study suggests that the relevance of sensory impairment to cognitive ageing may begin before clinical dementia becomes apparent. Compared with endpoint analyses that focus only on dementia, identifying normal-to-CIND deterioration and reduced CIND-to-normal recovery may better define the window in which sensory-health assessment and intervention could be evaluated. Recent randomized evidence from hearing-intervention trials provides important clues about whether sensory-health interventions can alter cognitive trajectories, although effects may depend on baseline risk, intervention timing, and follow-up duration (Lin et al., 2023). Future studies should combine objective visual and hearing measures, information on hearing aids and vision correction, and longer follow-up to test whether improving sensory function can alter early cognitive-state transitions.

## Conclusion

In a death-inclusive multistate Markov analysis of HRS data, visual, hearing, and dual sensory impairment were associated with increased transition from normal cognition to CIND, and visual and dual sensory impairment were associated with reduced recovery from CIND to normal cognition. Associations with dementia progression and mortality transitions were less consistent. These results suggest that sensory impairment, especially dual sensory impairment, is most strongly linked to early cognitive vulnerability and reduced recovery in later life.

## Data Availability

The original contributions presented in the study are included in the article/Supplementary material, further inquiries can be directed to the corresponding author.
