# Supplementary material for: Sensory impairment and cognitive-state transitions in middle-aged and older adults: a multistate Markov analysis of the health and retirement study
Source: Front Aging Neurosci. 2026 Sep 7;18:1913857. doi: 10.3389/fnagi.2026.1913857 (PMC13590718; doi:10.3389/fnagi.2026.1913857)
Supplement: Supplementary file 1 [file Data_Sheet_1.PDF]

Supplementary Materials

Sensory impairment and cognitive-state transitions in middle-aged and older adults: a multistate Markov analysis of the Health and Retirement Study

Supplementary Methods

Sensitivity analyses examined whether the primary findings were robust to alternative exposure definitions and model specifications. These included time-varying sensory impairment, lagged sensory impairment, restriction to baseline-normal participants, exclusion of baseline dementia, stricter sensory impairment definitions, an absorbing dementia assumption, refined death timing, an expanded transition-structure check allowing normal cognition-to-dementia intervals, complete-case inverse-probability weighting, observed spell-duration adjustment, and expanded adjustment for lifestyle and social-participation covariates.

Covariate missingness and baseline characteristics of participants included in and excluded from the complete-case adjusted model were summarized to assess potential selection. Baseline age and education distributions were compared across sensory groups using one-way analysis of variance and Pearson's chi-square test. The expanded-confounding model additionally included baseline current smoking, any alcohol use, weekly moderate or vigorous physical activity, and regular religious participation at least two or three times per month as an available but partial proxy for social engagement. This analysis included 15,936 participants. A core model was refitted in the same sample so that changes in estimates could be attributed to covariate adjustment rather than sample restriction.

Exploratory subgroup analyses used transition-specific logistic models stratified by age group, sex, and education and were treated as hypothesis-generating. These models were adjusted within strata for the remaining core covariates where applicable. They were not formal interaction tests; differences in statistical significance between strata should not be interpreted as evidence of effect modification.

Restricted expected time in each cognitive and mortality state was estimated by integrating model-based state occupation probabilities from the primary adjusted multistate model. Time-varying sensory status was updated at each available HRS wave using wave-specific self-rated vision and hearing; wave 15 sensory status was carried forward to wave 16 because direct sensory variables were not available for wave 16 in the local HRS sensory file. A lagged exposure model used sensory status from the previous wave, with baseline sensory status used when no prior wave was available.

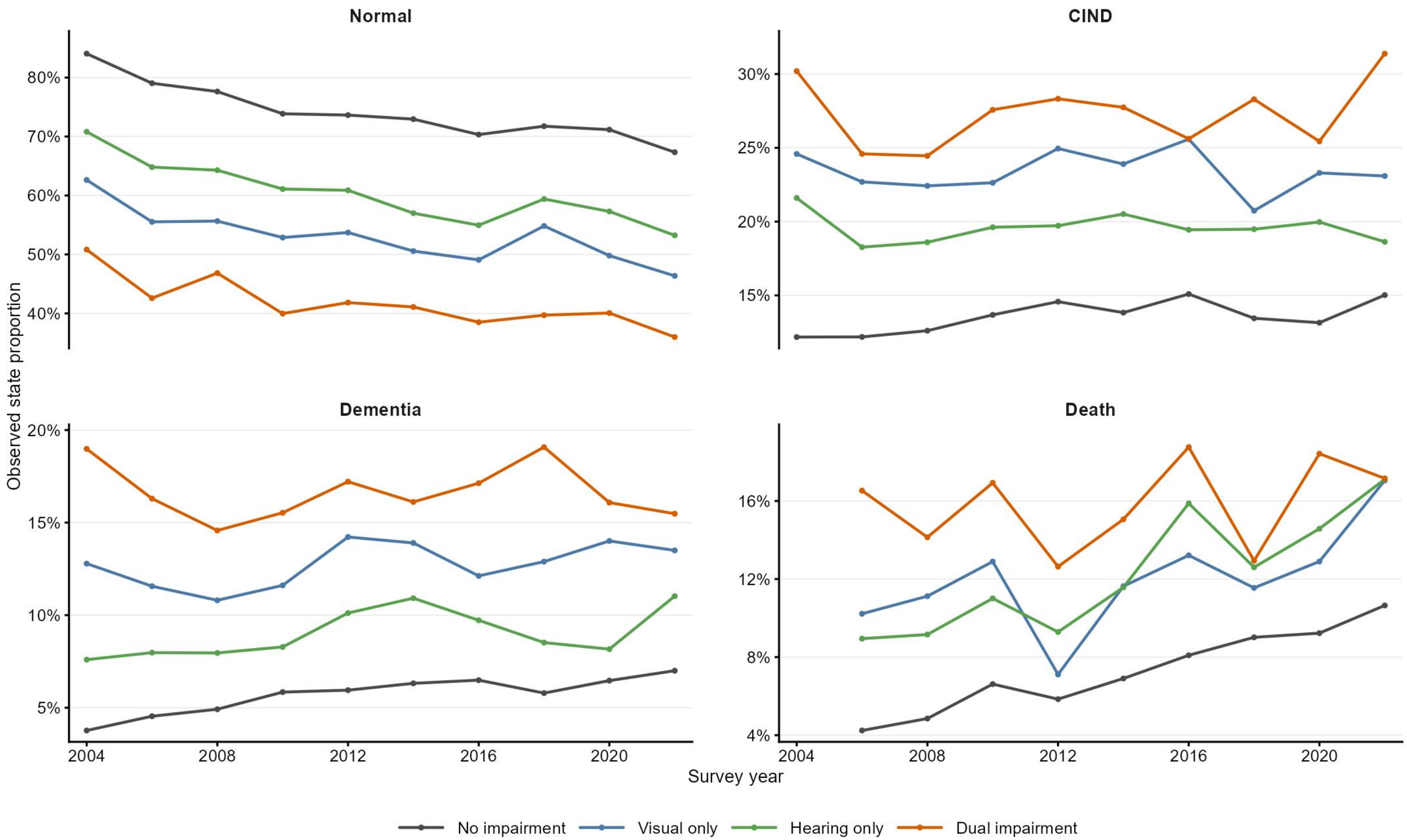

Supplementary Figure 1. Observed state occupation by baseline sensory group. Observed proportions of participants in each state are shown over survey years by baseline sensory impairment group. These empirical curves are descriptive and are not adjusted for covariates.

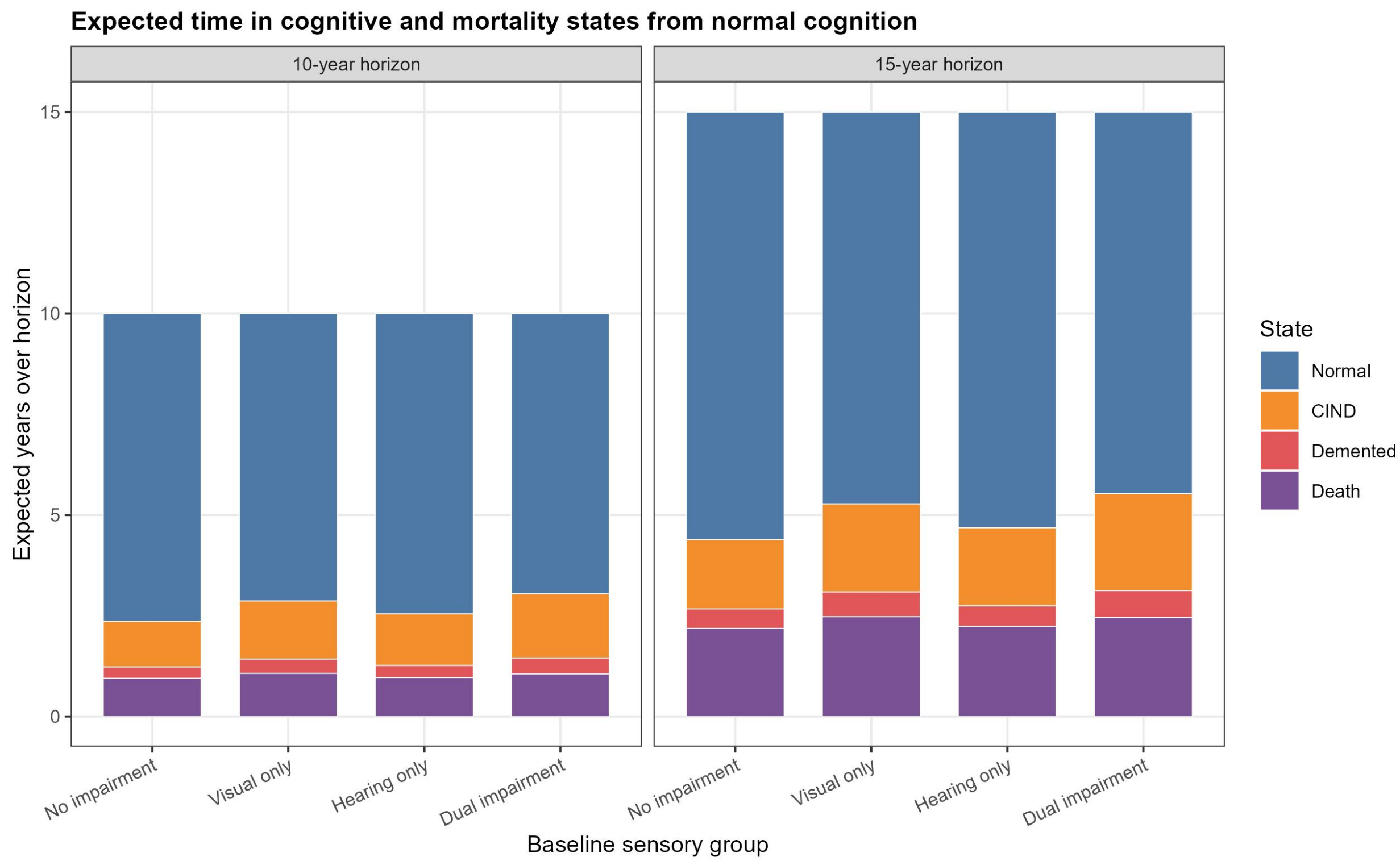

Supplementary Figure 2. Expected time in cognitive and mortality states from normal cognition. Restricted expected time was estimated by integrating model-based state occupation probabilities over 10- and 15-year horizons.

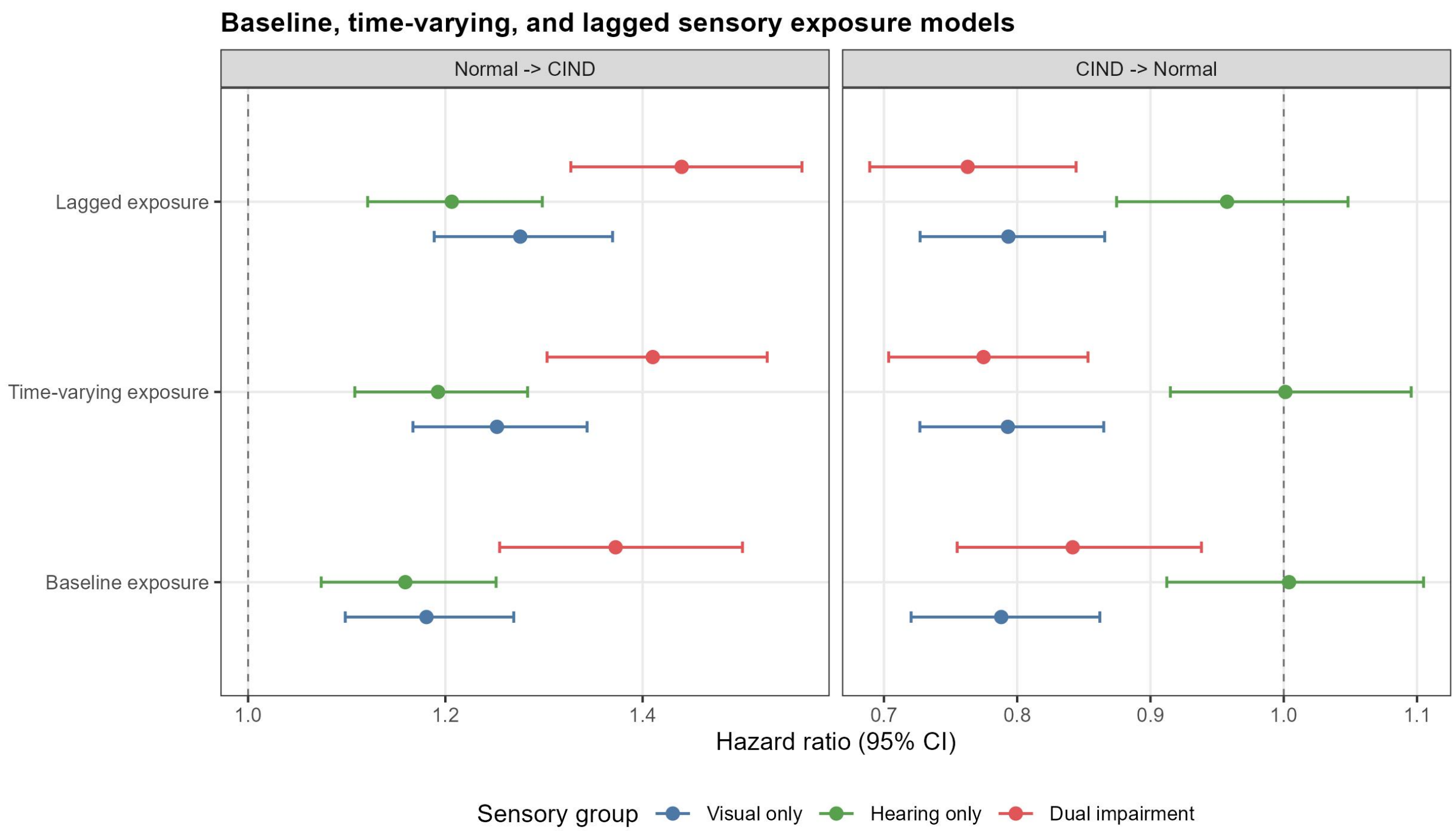

Supplementary Figure 3. Baseline, time-varying, and lagged sensory exposure models for early cognitive transitions. Forest plots compare hazard ratios and 95% confidence intervals for normal cognition to CIND and CIND to normal cognition.

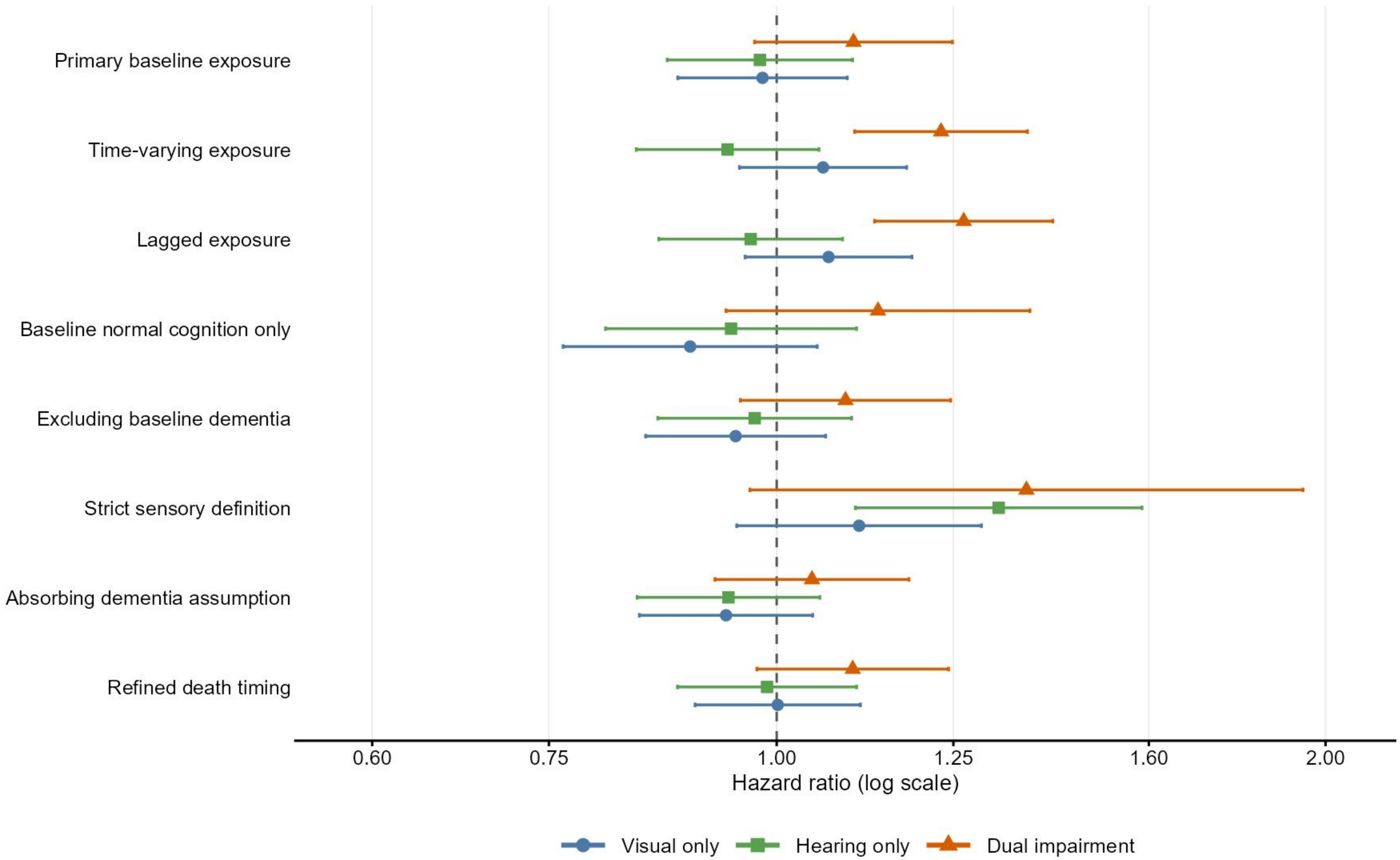

Supplementary Figure 4. Sensitivity analyses for CIND to dementia progression. Forest plot shows sensitivity analyses for progression from CIND to dementia. Compared with the early cognitive transition results, estimates for dementia progression were less consistent across model specifications.

Supplementary Tables

Supplementary Table 1. Observed transition counts.

From state	To state	Observed count
Normal	Normal	64079
CIND	Normal	5628
Dementia	Normal	317
Normal	CIND	8589
CIND	CIND	7135
Dementia	CIND	1273
Normal	Dementia	1163
CIND	Dementia	2940
Dementia	Dementia	3887
Normal	Death	3561
CIND	Death	2377
Dementia	Death	2803

Counts are descriptive and represent observed transitions in the constructed multistate dataset.

Supplementary Table 2. Covariate missingness in the analytic multistate dataset.

Variable	Missing, n (%)
age	0 (0.0)
sex	0 (0.0)
education	0 (0.0)
wealth_tertile	651 (3.6)
cesd	1618 (8.8)
chronic_count	0 (0.0)
sensory	0 (0.0)

Missingness was summarized among participants in the constructed analytic multistate dataset before complete-case restriction.

Supplementary Table 3. Baseline characteristics by complete-case inclusion status.

Complete-case status	N	Age, mean (SD)	Female, n (%)	Lower education, n (%)	CESD, mean (SD)	Chronic conditions, mean (SD)	Normal cognition, n (%)	CIND, n (%)	Dementia, n (%)	No impairment, n (%)	Visual only, n (%)	Hearing only, n (%)	Dual impairment, n (%)
Included in complete-case adjusted model	16083	67.4 (10.4)	9524 (59.2)	3425 (21.3)	1.4 (1.9)	1.9 (1.4)	12840 (79.8)	2603 (16.2)	640 (4.0)	10628 (66.1)	2104 (13.1)	2035 (12.7)	1316 (8.2)
Excluded from complete-case adjusted model	2209	69.7 (12.6)	1037 (46.9)	888 (40.2)	2.8 (2.5)	2.4 (1.6)	1109 (50.2)	471 (21.3)	629 (28.5)	1048 (47.4)	430 (19.5)	335 (15.2)	396 (17.9)

Values are shown as mean (SD) or n (%). Excluded participants had at least one missing covariate required for the primary adjusted model.

Supplementary Table 4. Restricted expected time in cognitive and mortality states from normal cognition.

Values are expected years over the specified horizon. Difference columns show absolute differences versus the no-impairment group within the same horizon.

Sensory group	Horizon, years	Normal	CIND	Dementia	Death	Diff Normal vs no impairment	Diff CIND vs no impairment	Diff Dementia vs no impairment	Diff Death vs no impairment
No impairment	10	7.64	1.14	0.28	0.95				
Visual only	10	7.13	1.44	0.35	1.07	-0.51	+0.31	+0.07	+0.12
Hearing only	10	7.45	1.28	0.30	0.97	-0.19	+0.15	+0.02	+0.02
Dual impairment	10	6.95	1.59	0.39	1.06	-0.68	+0.46	+0.12	+0.11
No impairment	15	10.61	1.72	0.48	2.19				
Visual only	15	9.73	2.19	0.61	2.47	-0.89	+0.47	+0.13	+0.29
Hearing only	15	10.32	1.94	0.51	2.24	-0.29	+0.22	+0.03	+0.05
Dual impairment	15	9.47	2.40	0.67	2.46	-1.14	+0.68	+0.18	+0.27

Supplementary Table 5. Sensitivity analyses for key cognitive transitions.

Model	Exposure	Transition	HR (95% CI)
Primary baseline exposure	Visual only	Normal → CIND	1.18 (1.10-1.27)
Primary baseline exposure	Visual only	CIND → Normal	0.79 (0.72-0.86)
Primary baseline exposure	Visual only	CIND → Dementia	0.98 (0.88-1.09)
Primary baseline exposure	Hearing only	Normal → CIND	1.16 (1.07-1.25)
Primary baseline exposure	Hearing only	CIND → Normal	1.00 (0.91-1.10)
Primary baseline exposure	Hearing only	CIND → Dementia	0.98 (0.87-1.10)
Primary baseline exposure	Dual impairment	Normal → CIND	1.37 (1.26-1.50)
Primary baseline exposure	Dual impairment	CIND → Normal	0.84 (0.75-0.94)
Primary baseline exposure	Dual impairment	CIND → Dementia	1.10 (0.97-1.25)
Time-varying exposure	Visual only	Normal → CIND	1.25 (1.17-1.34)
Time-varying exposure	Visual only	CIND → Normal	0.79 (0.73-0.86)
Time-varying exposure	Visual only	CIND → Dementia	1.06 (0.95-1.18)
Time-varying exposure	Hearing only	Normal → CIND	1.19 (1.11-1.28)
Time-varying exposure	Hearing only	CIND → Normal	1.00 (0.91-1.10)
Time-varying exposure	Hearing only	CIND → Dementia	0.94 (0.84-1.05)
Time-varying exposure	Dual impairment	Normal → CIND	1.41 (1.30-1.53)
Time-varying exposure	Dual impairment	CIND → Normal	0.77 (0.70-0.85)
Time-varying exposure	Dual impairment	CIND → Dementia	1.23 (1.10-1.37)
Lagged exposure	Visual only	Normal → CIND	1.28 (1.19-1.37)
Lagged exposure	Visual only	CIND → Normal	0.79 (0.73-0.87)
Lagged exposure	Visual only	CIND → Dementia	1.07 (0.96-1.19)
Lagged exposure	Hearing only	Normal → CIND	1.21 (1.12-1.30)
Lagged exposure	Hearing only	CIND → Normal	0.96 (0.87-1.05)
Lagged exposure	Hearing only	CIND → Dementia	0.97 (0.86-1.09)
Lagged exposure	Dual impairment	Normal → CIND	1.44 (1.33-1.56)
Lagged exposure	Dual impairment	CIND → Normal	0.76 (0.69-0.84)
Lagged exposure	Dual impairment	CIND → Dementia	1.27 (1.13-1.42)
Baseline normal cognition only	Visual only	Normal → CIND	1.21 (1.11-1.32)
Baseline normal cognition only	Visual only	CIND → Normal	0.91 (0.81-1.02)
Baseline normal cognition only	Visual only	CIND → Dementia	0.90 (0.76-1.05)

Model	Exposure	Transition	HR (95% CI)
Baseline normal cognition only	Hearing only	Normal → CIND	1.16 (1.06-1.27)
Baseline normal cognition only	Hearing only	CIND → Normal	1.07 (0.95-1.21)
Baseline normal cognition only	Hearing only	CIND → Dementia	0.94 (0.81-1.11)
Baseline normal cognition only	Dual impairment	Normal → CIND	1.37 (1.23-1.52)
Baseline normal cognition only	Dual impairment	CIND → Normal	0.91 (0.78-1.05)
Baseline normal cognition only	Dual impairment	CIND → Dementia	1.14 (0.94-1.38)
Excluding baseline dementia	Visual only	Normal → CIND	1.18 (1.10-1.27)
Excluding baseline dementia	Visual only	CIND → Normal	0.81 (0.74-0.89)
Excluding baseline dementia	Visual only	CIND → Dementia	0.95 (0.85-1.06)
Excluding baseline dementia	Hearing only	Normal → CIND	1.17 (1.08-1.26)
Excluding baseline dementia	Hearing only	CIND → Normal	1.02 (0.93-1.13)
Excluding baseline dementia	Hearing only	CIND → Dementia	0.97 (0.86-1.10)
Excluding baseline dementia	Dual impairment	Normal → CIND	1.38 (1.26-1.51)
Excluding baseline dementia	Dual impairment	CIND → Normal	0.85 (0.76-0.95)
Excluding baseline dementia	Dual impairment	CIND → Dementia	1.09 (0.96-1.25)
Strict sensory definition	Visual only	Normal → CIND	1.09 (0.97-1.24)
Strict sensory definition	Visual only	CIND → Normal	0.77 (0.66-0.90)
Strict sensory definition	Visual only	CIND → Dementia	1.11 (0.95-1.30)
Strict sensory definition	Hearing only	Normal → CIND	1.21 (1.06-1.37)
Strict sensory definition	Hearing only	CIND → Normal	1.15 (0.98-1.35)
Strict sensory definition	Hearing only	CIND → Dementia	1.32 (1.10-1.59)
Strict sensory definition	Dual impairment	Normal → CIND	1.12 (0.82-1.51)
Strict sensory definition	Dual impairment	CIND → Normal	1.05 (0.73-1.50)
Strict sensory definition	Dual impairment	CIND → Dementia	1.37 (0.97-1.94)
Absorbing dementia assumption	Visual only	Normal → CIND	1.17 (1.08-1.26)
Absorbing dementia assumption	Visual only	CIND → Normal	0.82 (0.74-0.90)
Absorbing dementia assumption	Visual only	CIND → Dementia	0.94 (0.84-1.05)
Absorbing dementia assumption	Hearing only	Normal → CIND	1.17 (1.08-1.27)
Absorbing dementia assumption	Hearing only	CIND → Normal	1.03 (0.93-1.14)
Absorbing dementia assumption	Hearing only	CIND → Dementia	0.94 (0.84-1.06)
Absorbing dementia assumption	Dual impairment	Normal → CIND	1.36 (1.24-1.50)

Model	Exposure	Transition	HR (95% CI)
Absorbing dementia assumption	Dual impairment	CIND → Normal	0.84 (0.75-0.94)
Absorbing dementia assumption	Dual impairment	CIND → Dementia	1.05 (0.93-1.18)
Refined death timing	Visual only	Normal → CIND	1.17 (1.09-1.26)
Refined death timing	Visual only	CIND → Normal	0.79 (0.72-0.87)
Refined death timing	Visual only	CIND → Dementia	1.00 (0.90-1.11)
Refined death timing	Hearing only	Normal → CIND	1.15 (1.07-1.24)
Refined death timing	Hearing only	CIND → Normal	1.01 (0.91-1.11)
Refined death timing	Hearing only	CIND → Dementia	0.99 (0.88-1.11)
Refined death timing	Dual impairment	Normal → CIND	1.37 (1.25-1.50)
Refined death timing	Dual impairment	CIND → Normal	0.84 (0.75-0.94)
Refined death timing	Dual impairment	CIND → Dementia	1.10 (0.98-1.24)
Complete-case IPCW weighted	Dual impairment	CIND → Dementia	1.12 (0.99-1.27)
Complete-case IPCW weighted	Dual impairment	CIND → Normal	0.83 (0.74-0.92)
Complete-case IPCW weighted	Dual impairment	Normal → CIND	1.37 (1.25-1.51)
Complete-case IPCW weighted	Hearing only	CIND → Dementia	0.99 (0.88-1.11)
Complete-case IPCW weighted	Hearing only	CIND → Normal	0.99 (0.90-1.09)
Complete-case IPCW weighted	Hearing only	Normal → CIND	1.16 (1.07-1.25)
Complete-case IPCW weighted	Visual only	CIND → Dementia	1.01 (0.91-1.12)
Complete-case IPCW weighted	Visual only	CIND → Normal	0.76 (0.70-0.84)
Complete-case IPCW weighted	Visual only	Normal → CIND	1.19 (1.10-1.28)
Observed duration-adjusted	Dual impairment	CIND → Dementia	1.06 (0.94-1.20)
Observed duration-adjusted	Dual impairment	CIND → Normal	0.89 (0.80-0.99)
Observed duration-adjusted	Dual impairment	Normal → CIND	1.35 (1.24-1.48)
Observed duration-adjusted	Hearing only	CIND → Dementia	0.99 (0.88-1.11)
Observed duration-adjusted	Hearing only	CIND → Normal	1.01 (0.92-1.12)
Observed duration-adjusted	Hearing only	Normal → CIND	1.13 (1.05-1.22)
Observed duration-adjusted	Visual only	CIND → Dementia	0.98 (0.88-1.09)
Observed duration-adjusted	Visual only	CIND → Normal	0.82 (0.75-0.90)
Observed duration-adjusted	Visual only	Normal → CIND	1.18 (1.10-1.27)

Rows show hazard ratios and 95% confidence intervals for selected key transitions across model specifications.

Supplementary Table 6. Baseline, time-varying, lagged, weighted, and duration-adjusted models for key cognitive transitions.

Rows show hazard ratios and 95% confidence intervals for selected transitions. IPCW denotes inverse-probability of complete-case weighting.

Model	Exposure	Transition	HR (95% CI)
Baseline exposure	Visual only	CIND → Dementia	0.98 (0.88-1.09)
Baseline exposure	Visual only	CIND → Normal	0.79 (0.72-0.86)
Baseline exposure	Visual only	Normal → CIND	1.18 (1.10-1.27)
Baseline exposure	Hearing only	CIND → Dementia	0.98 (0.87-1.10)
Baseline exposure	Hearing only	CIND → Normal	1.00 (0.91-1.10)
Baseline exposure	Hearing only	Normal → CIND	1.16 (1.07-1.25)
Baseline exposure	Dual impairment	CIND → Dementia	1.10 (0.97-1.25)
Baseline exposure	Dual impairment	CIND → Normal	0.84 (0.75-0.94)
Baseline exposure	Dual impairment	Normal → CIND	1.37 (1.26-1.50)
Time-varying exposure	Visual only	CIND → Dementia	1.06 (0.95-1.18)
Time-varying exposure	Visual only	CIND → Normal	0.79 (0.73-0.86)
Time-varying exposure	Visual only	Normal → CIND	1.25 (1.17-1.34)
Time-varying exposure	Hearing only	CIND → Dementia	0.94 (0.84-1.05)
Time-varying exposure	Hearing only	CIND → Normal	1.00 (0.91-1.10)
Time-varying exposure	Hearing only	Normal → CIND	1.19 (1.11-1.28)
Time-varying exposure	Dual impairment	CIND → Dementia	1.23 (1.10-1.37)
Time-varying exposure	Dual impairment	CIND → Normal	0.77 (0.70-0.85)
Time-varying exposure	Dual impairment	Normal → CIND	1.41 (1.30-1.53)
Lagged exposure	Visual only	CIND → Dementia	1.07 (0.96-1.19)
Lagged exposure	Visual only	CIND → Normal	0.79 (0.73-0.87)
Lagged exposure	Visual only	Normal → CIND	1.28 (1.19-1.37)
Lagged exposure	Hearing only	CIND → Dementia	0.97 (0.86-1.09)
Lagged exposure	Hearing only	CIND → Normal	0.96 (0.87-1.05)
Lagged exposure	Hearing only	Normal → CIND	1.21 (1.12-1.30)
Lagged exposure	Dual impairment	CIND → Dementia	1.27 (1.13-1.42)
Lagged exposure	Dual impairment	CIND → Normal	0.76 (0.69-0.84)
Lagged exposure	Dual impairment	Normal → CIND	1.44 (1.33-1.56)
Complete-case IPCW weighted	Visual only	CIND → Dementia	1.01 (0.91-1.12)
Complete-case IPCW weighted	Visual only	CIND → Normal	0.76 (0.70-0.84)
Complete-case IPCW weighted	Visual only	Normal → CIND	1.19 (1.10-1.28)
Complete-case IPCW weighted	Hearing only	CIND → Dementia	0.99 (0.88-1.11)
Complete-case IPCW weighted	Hearing only	CIND → Normal	0.99 (0.90-1.09)
Complete-case IPCW weighted	Hearing only	Normal → CIND	1.16 (1.07-1.25)
Complete-case IPCW weighted	Dual impairment	CIND → Dementia	1.12 (0.99-1.27)
Complete-case IPCW weighted	Dual impairment	CIND → Normal	0.83 (0.74-0.92)
Complete-case IPCW weighted	Dual impairment	Normal → CIND	1.37 (1.25-1.51)
Observed duration-adjusted	Visual only	CIND → Dementia	0.98 (0.88-1.09)
Observed duration-adjusted	Visual only	CIND → Normal	0.82 (0.75-0.90)
Observed duration-adjusted	Visual only	Normal → CIND	1.18 (1.10-1.27)
Observed duration-adjusted	Hearing only	CIND → Dementia	0.99 (0.88-1.11)
Observed duration-adjusted	Hearing only	CIND → Normal	1.01 (0.92-1.12)
Observed duration-adjusted	Hearing only	Normal → CIND	1.13 (1.05-1.22)
Observed duration-adjusted	Dual impairment	CIND → Dementia	1.06 (0.94-1.20)
Observed duration-adjusted	Dual impairment	CIND → Normal	0.89 (0.80-0.99)
Observed duration-adjusted	Dual impairment	Normal → CIND	1.35 (1.24-1.48)

Supplementary Table 7. Time-varying sensory impairment distribution by HRS wave.

Values are n (%). Wave 15 sensory status was carried forward to wave 16.

Wave	Year	No impairment	Visual only	Hearing only	Dual impairment
7	2004	10,628 (66.1%)	2,104 (13.1%)	2,035 (12.7%)	1,316 (8.2%)
8	2006	10,275 (63.9%)	2,098 (13.0%)	2,177 (13.5%)	1,533 (9.5%)
9	2008	9,446 (64.1%)	1,970 (13.4%)	1,924 (13.1%)	1,390 (9.4%)
10	2010	8,407 (63.2%)	1,745 (13.1%)	1,801 (13.5%)	1,356 (10.2%)
11	2012	7,354 (62.4%)	1,478 (12.5%)	1,639 (13.9%)	1,324 (11.2%)
12	2014	6,503 (61.5%)	1,463 (13.8%)	1,450 (13.7%)	1,164 (11.0%)
13	2016	5,544 (60.4%)	1,250 (13.6%)	1,371 (14.9%)	1,017 (11.1%)
14	2018	4,601 (62.4%)	937 (12.7%)	1,033 (14.0%)	799 (10.8%)
15	2020	3,945 (63.8%)	778 (12.6%)	826 (13.4%)	631 (10.2%)
16	2022	3,158 (63.9%)	618 (12.5%)	665 (13.4%)	505 (10.2%)

Supplementary Table 8. Person-level time-varying sensory impairment patterns.

Patterns were summarized among participants in the complete-case adjusted sample.

Pattern	N	%
Persistently no impairment	5,433	33.8
Incident sensory impairment	5,195	32.3
Persistent/progressive dual component	2,623	16.3
Persistent or changing single impairment	2,031	12.6
Improved to no impairment	801	5.0

Supplementary Table 9. Expanded transition-structure check allowing Normal to Dementia intervals.

Exposure	Transition	HR point estimate	Inference note
Visual only	Normal → CIND	1.18	Expanded q-matrix sensitivity; Hessian non-positive, so formal confidence intervals were not used.
Visual only	Normal → Dementia	1.05	Expanded q-matrix sensitivity; Hessian non-positive, so formal confidence intervals were not used.
Visual only	CIND → Normal	0.79	Expanded q-matrix sensitivity; Hessian non-positive, so formal confidence intervals were not used.
Visual only	CIND → Dementia	0.98	Expanded q-matrix sensitivity; Hessian non-positive, so formal confidence intervals were not used.
Hearing only	Normal → CIND	1.16	Expanded q-matrix sensitivity; Hessian non-positive, so formal confidence intervals were not used.
Hearing only	Normal → Dementia	1.28	Expanded q-matrix sensitivity; Hessian non-positive, so formal confidence intervals were not used.
Hearing only	CIND → Normal	1.00	Expanded q-matrix sensitivity; Hessian non-positive, so formal confidence intervals were not used.
Hearing only	CIND → Dementia	0.98	Expanded q-matrix sensitivity; Hessian non-positive, so formal confidence intervals were not used.
Dual impairment	Normal → CIND	1.37	Expanded q-matrix sensitivity; Hessian non-positive, so formal confidence intervals were not used.
Dual impairment	Normal → Dementia	1.71	Expanded q-matrix sensitivity; Hessian non-positive, so formal confidence intervals were not used.
Dual impairment	CIND → Normal	0.84	Expanded q-matrix sensitivity; Hessian non-positive, so formal confidence intervals were not used.
Dual impairment	CIND → Dementia	1.10	Expanded q-matrix sensitivity; Hessian non-positive, so formal confidence intervals were not used.

The expanded q-matrix sensitivity generated a non-positive Hessian; estimates are reported as point estimates only and were not used for formal inference.

Supplementary Table 10. Time-homogeneity assessment.

Model	-2 log likelihood	Interpretation
Time-homogeneous model	141,549.7	Primary estimates interpreted as average transition-specific associations over follow-up.
Piecewise model	141,265.0	Lower -2 log likelihood suggests transition intensities may vary over follow-up.

Supplementary Table 11. Exploratory subgroup analyses.

Transition	Subgroup	Level	Exposure	OR (95% CI)	P value	N
Normal → CIND	age_group	<75	Visual only	1.28 (1.18-1.39)	<0.001	62839
Normal → CIND	age_group	<75	Hearing only	1.17 (1.07-1.28)	<0.001	62839
Normal → CIND	age_group	<75	Dual impairment	1.50 (1.34-1.67)	<0.001	62839
CIND → Normal	age_group	<75	Visual only	0.73 (0.65-0.82)	<0.001	11128
CIND → Normal	age_group	<75	Hearing only	0.97 (0.85-1.11)	0.661	11128
CIND → Normal	age_group	<75	Dual impairment	0.72 (0.63-0.83)	<0.001	11128
Normal → CIND	age_group	>=75	Visual only	1.08 (0.92-1.27)	0.354	9049
Normal → CIND	age_group	>=75	Hearing only	1.05 (0.91-1.21)	0.481	9049
Normal → CIND	age_group	>=75	Dual impairment	1.14 (0.94-1.38)	0.182	9049
CIND → Normal	age_group	>=75	Visual only	0.85 (0.68-1.08)	0.179	4830
CIND → Normal	age_group	>=75	Hearing only	1.04 (0.85-1.27)	0.721	4830
CIND → Normal	age_group	>=75	Dual impairment	1.02 (0.80-1.31)	0.853	4830
Normal → CIND	sex	Male	Visual only	1.22 (1.07-1.40)	0.003	28504
Normal → CIND	sex	Male	Hearing only	1.16 (1.05-1.29)	0.003	28504
Normal → CIND	sex	Male	Dual impairment	1.47 (1.28-1.69)	<0.001	28504
CIND → Normal	sex	Male	Visual only	0.74 (0.62-0.88)	<0.001	6445
CIND → Normal	sex	Male	Hearing only	0.98 (0.85-1.13)	0.754	6445
CIND → Normal	sex	Male	Dual impairment	0.84 (0.71-1.00)	0.054	6445
Normal → CIND	sex	Female	Visual only	1.26 (1.15-1.38)	<0.001	43384
Normal → CIND	sex	Female	Hearing only	1.11 (0.99-1.25)	0.065	43384
Normal → CIND	sex	Female	Dual impairment	1.31 (1.15-1.49)	<0.001	43384
CIND → Normal	sex	Female	Visual only	0.74 (0.65-0.83)	<0.001	9513
CIND → Normal	sex	Female	Hearing only	1.00 (0.85-1.17)	0.971	9513
CIND → Normal	sex	Female	Dual impairment	0.72 (0.61-0.85)	<0.001	9513
Normal → CIND	edu_group	lower	Visual only	1.29 (1.18-1.41)	<0.001	33972
Normal → CIND	edu_group	lower	Hearing only	1.13 (1.02-1.24)	0.014	33972
Normal → CIND	edu_group	lower	Dual impairment	1.40 (1.25-1.56)	<0.001	33972
CIND → Normal	edu_group	lower	Visual only	0.74 (0.66-0.83)	<0.001	11983
CIND → Normal	edu_group	lower	Hearing only	1.02 (0.90-1.16)	0.720	11983
CIND → Normal	edu_group	lower	Dual impairment	0.79 (0.69-0.91)	<0.001	11983
Normal → CIND	edu_group	higher	Visual only	1.12 (0.97-1.29)	0.137	37916

Transition	Subgroup	Level	Exposure	OR (95% CI)	P value	N
Normal → CIND	edu_group	higher	Hearing only	1.17 (1.03-1.32)	0.013	37916
Normal → CIND	edu_group	higher	Dual impairment	1.32 (1.10-1.59)	0.003	37916
CIND → Normal	edu_group	higher	Visual only	0.78 (0.61-0.98)	0.032	3975
CIND → Normal	edu_group	higher	Hearing only	0.87 (0.71-1.07)	0.179	3975
CIND → Normal	edu_group	higher	Dual impairment	0.72 (0.55-0.95)	0.019	3975

ORs were estimated using transition-specific logistic models. Models were adjusted for age, sex, education, wealth tertile, depressive symptoms, and chronic disease count, excluding the stratification variable where applicable. These analyses are exploratory stratified estimates rather than formal interaction tests; differences in statistical significance between strata do not establish effect modification.

Supplementary Table 12. Expanded adjustment for lifestyle factors and regular religious participation.

Transition	Sensory impairment group	Same-sample core-adjusted HR (95% CI)	Expanded-adjusted HR (95% CI)
Normal cognition to CIND	Visual only	1.19 (1.10-1.27)	1.17 (1.09-1.26)
Normal cognition to CIND	Hearing only	1.16 (1.07-1.25)	1.16 (1.08-1.26)
Normal cognition to CIND	Dual impairment	1.37 (1.25-1.50)	1.35 (1.23-1.48)
CIND to normal cognition	Visual only	0.80 (0.73-0.87)	0.80 (0.73-0.88)
CIND to normal cognition	Hearing only	1.01 (0.91-1.11)	1.00 (0.91-1.10)
CIND to normal cognition	Dual impairment	0.84 (0.75-0.94)	0.84 (0.76-0.94)
CIND to dementia	Visual only	0.99 (0.89-1.10)	0.98 (0.88-1.09)
CIND to dementia	Hearing only	0.97 (0.87-1.10)	0.97 (0.86-1.09)
CIND to dementia	Dual impairment	1.12 (0.98-1.27)	1.11 (0.98-1.26)

HRs compare each sensory impairment group with no sensory impairment among 15,936 participants with complete data for all expanded covariates. The core-adjusted model included age, sex, education, wealth tertile, depressive symptoms, and chronic disease count. The expanded model additionally included current smoking, any alcohol use, weekly moderate or vigorous physical activity, and regular religious participation. Regular religious participation is an incomplete proxy for social engagement.
